# Substance use during pregnancy: the role of mindfulness in reducing stigma

**DOI:** 10.3389/fpsyg.2024.1432926

**Published:** 2024-06-25

**Authors:** Taylor M. Shank, Surja Tjahaja, Tara M. Rutter, Kristen L. Mackiewicz Seghete

**Affiliations:** ^1^Department of Psychiatry and Neurology, Oregon Health and Science University, Portland, OR, United States; ^2^MindfulnessMeditation.us, Portland, OR, United States; ^3^Department of Psychiatry, Department of Obstetrics and Gynecology, and Center for Mental Health Innovation, Oregon Health and Science University, Portland, OR, United States

**Keywords:** perinatal substance use, stigma, mindfulness, pregnancy, Buddhism, perinatal, bias

## Abstract

Stigmatization is a significant healthcare barrier among individuals who utilize substances during pregnancy. Of the 3.6 million U.S. births each year, approximately 10% are affected by perinatal substance use, an estimate which is likely underestimated due to fear of stigma and prosecution. Of those experiencing perinatal substance use, less than 11% receive treatment, while maternal deaths due to overdose during the postpartum period have increased by 81% from 2017 to 2020. Societal perception of non-pregnant individuals experiencing substance use disorders recognizes the biological basis of addiction, whereas for pregnant individuals, societal perception slides into moral failing as the basis of addiction. Many recommendations and guidelines for decreasing substance use stigmatization among non-pregnant and pregnant individuals exist. We focus on the use of mindfulness in recognizing and addressing structural and social stigma within healthcare systems. Mindfulness has been extracted from its roots as an essential element of the Eightfold Path in Buddhism, which largely centers on living ethically to reduce suffering of self and others. By acknowledging the roots of mindfulness, providers can engage mindfully in practices that help identify one’s overarching personal values and encourage one to lead healthcare encounters compassion and willingness to support help-seeking community members who are experiencing suffering. A deeper awareness of mindfulness practices within the context of ethical conduct can support healthcare shifts away from criminalization toward more patient- and family-centered approaches.

## Perinatal substance use

Recent United States data suggests 10–11% of individuals use substances during pregnancy (Denny et al., 2019; England et al., 2020). From 2017 to 2020, substance overdose during pregnancy and postpartum increased by 81% (Bruzelius and Martins, 2022), making overdose a leading cause of maternal death (Trost et al., 2022). While alcohol is the most common substance used by pregnant individuals (Substance Abuse and Mental Health Services Administration Department of Health and Human Services, 2019), opioid use is significantly increasing as our country faces an opioid epidemic (Ko et al., 2020). Rates of opioid-related diagnoses documented at delivery increased by 131% from 2010 to 2017, and one in five reported eventual misuse (Ko et al., 2020). With such drastic increases in perinatal opioid use, it is startling that pregnant patients attempting to engage in treatment are 17% less likely than non-pregnant patients to be granted appointments when calling into opioid treatment centers (Patrick et al., 2020). Overall, in 2019, less than 11% of individuals experiencing perinatal substance use received treatment (Substance Abuse and Mental Health Services Administration Department of Health and Human Services, 2019).

As nearly half of U.S. states criminalize perinatal substance use (Weber et al., 2021), current prevalence rates are likely underrepresented due to fear of stigma and prosecution (Howard, 2015). Providers are hesitant to treat perinatal substance use, as limited research perpetuates provider doubt and discomfort (Webb et al., 2021). Increased ethical challenges of evaluating medication-based treatments for opioid use during pregnancy further complicate the issue. Additionally, pregnant individuals using substances are often excluded from perinatal clinical research, in particular clinical trials of effective interventions for co-occurring mental health conditions (Mackiewicz Seghete et al., 2020). Traditional research and policy have focused disproportionately on the effects of substance use on the developing fetus compared to maternal risk, which contributes to limited support for perinatal individuals and increased shame around utilization of substances (Mackiewicz Seghete et al., 2020). This multitude of factors leaves individuals experiencing perinatal substance use marginalized and oppressed, both specifically within systems of care and more broadly within society.

## How stigma impacts perinatal substance use

As several authors (e.g., Nichols et al., 2021; Weber et al., 2021) have thoroughly discussed the complex effects of substance-related stigma on birthing persons and infants, we will build upon the understanding of stigma, how stigma manifests interpersonally, and how mindfulness can support conscious awareness of the counterproductive effects of stigma. Stigma is a dynamic and interpersonal process operating on many levels, including structural, social, and internalized levels (Phelan et al., 2008). As language usage and societal messaging shifts (e.g., drug addict vs. person with a substance use disorder), social perception changes and stigma can be greatly reduced. Research indicates that societal perspectives of those with substance dependence have largely shifted from beliefs rooted in “moral failing” to an understanding that substance dependence is a “chronic relapsing brain disorder” (National Institute on Drug Abuse, 2016) marked by intense urges to cope via self-medication (Khantzian, 1997). However, with regard to pregnant persons using substances, societal beliefs revert toward “moral failing” (Racine et al., 2015). Societally, more judgment is held toward pregnant individuals due to idealized narratives of motherhood and stringent beliefs that perinatal substance use signifies complete “maternal unfitness” (Terplan et al., 2015).

Structural stigma, which is stigma embedded in institutional policies, cultural norms, and laws (Lipsett et al., 2023), results in healthcare practices that condemn—rather than support—pregnant substance users. Those who experience systemic oppression and discrimination due to racialization and minoritization are at greater risk of stigmatization, as perinatal substance use can be weaponized to portray parental unfitness and involve child protective services (Terplan et al., 2015). In fact, children exposed to substances during pregnancy who are born to Black parents are four times more likely to be reported to child protective services (Kerker et al., 2006; Roberts and Nuru-Jeter, 2012 as cited by Nichols et al., 2021). Racialized pregnant persons and low-income pregnant persons systematically receive harsher legal consequences for substance use (Kennedy-Hendricks et al., 2016; Terplan et al., 2015; Bridges, 2020 as cited by Nichols et al., 2021). Criminalization is intended to reduce perinatal substance use and poor infant outcomes, such as neonatal withdrawal syndrome, but in reality, criminalization only reduces access to and engagement with healthcare (Weber et al., 2021). A 2015 qualitative study reported 73.3% of participants feared being identified as a substance user, as they could lose custody of their children or face criminal penalties (Stone, 2015). The study further stated the primary strategy for preventing detection was medical care avoidance, often skipping prenatal care altogether (Stone, 2015).

Social stigma exists between individuals, within interpersonal interactions. Many individuals experiencing perinatal substance use report experiencing discrimination, micro-aggressions, stereotyping, and sub-par care as a result of personally-held beliefs of healthcare providers, such as being called “junkie” or “bad mother” (Harvey et al., 2015; Howard, 2015; Blair et al., 2021) and being turned away from treatment (Burgess et al., 2021). Bright et al. (2022) succinctly summarize the troubling dilemma many pregnant substance users face: “Stigma prevents them from seeking care, and stigma about care received decreases treatment motivation” (Bright et al., 2022). Limited investment in perinatal substance use research and treatment, due to structural stigma, reinforces interpersonal stigma. While both are intended to function as practices that compel individuals to abide by cultural norms, they actually impact individuals by increasing internalized stigma and shame (Lipsett et al., 2023).

Internalized stigma is the infiltration of society’s beliefs into one’s self-perception, where an individual takes on and identifies with the stigmatizing belief. Shame is the emotional core of stigma (Hill and Leeming, 2014 as cited by Earnshaw, 2020), and often fosters resignation (Burgess et al., 2021). One qualitative analysis showed several participants reporting, “People judge you. Why try to do your best to stay clean when you get no support?” and “If you are being accused of doing things you might as well just do it” (Burgess et al., 2021). Internalized shame and helplessness are highly likely to increase substance use as a means of coping with unbearable internal experiences (see Self-Medication Theory, Khantzian, 1997).

## Importance of provider-facing tools for reducing stigma

Stigma does not emerge within a vacuum of a single individual’s mind. It is human nature to categorize and to establish and abide by hierarchy. Based on the work of Goffman (1963), *stigma* dates back to the Greeks, and was a method of devaluation of those believed to have a “spoiled social identity” (Goffman, 1963 as cited by Bos et al., 2013). As described by National Institute on Drug Abuse (2016), the overwhelming power of substance use compulsions is almost inconceivable to individuals without an experience of substance dependence, as such, it is easy to overestimate the access to self-control one “should” have in relation to substance addiction (National Institute on Drug Abuse, 2016, pg. 51). Conditioned beliefs that individuals can and should “just stop” using substances because they have become pregnant become unconsciously regarded as basic truths despite scientific knowledge that substance addiction is signified by difficulty controlling substance use and preventing relapse. When perinatal substance use is viewed as a moral failing or a “deviant choice, rather than a medical disorder” (Volkow, 2023), the high level of perceived personal responsibility generates anger (Bos et al., 2013) and an underlying sense these individuals are undeserving of help because they made the choice to use substances (Volkow, 2023). Thus, a lack of understanding regarding the power of substance use breeds an implicit “othering.”

As summarized by Akbulut and Razum (2022), othering is an embedded social process geared toward identifying those who do not belong. Othering results in disempowerment, marginalization, and disproportionate access to resources (Akbulut and Razum, 2022). Othering also breeds distancing from and fear of those engaging in the stigmatized behavior, as they are believed to be dangerous to others (Bos et al., 2013). Anger and fear toward individuals engaged in perinatal substance use can create “compassion fatigue” in healthcare providers, marked by lowered empathy and lack of respect toward stigmatized patients (Sweigart, 2017). Qualitative field notes express how healthcare providers within the NICU who hold moral judgments toward perinatal substance use, provide less information to parents of infants experiencing withdrawal, as they are viewed as uncaring and unmotivated (Nichols et al., 2021). Treating patients differently based on personal intolerance infringes upon one’s ethical responsibility to provide equitable, empowering care to all patients.

While stigma may be understood cognitively by providers, registering stigma in one’s own behavior can be difficult, as the behavior likely feels justified and has been culturally-validated. Current practices for anti-stigma education are not enough. Implicit bias training is often presented in an information-based single-session focused on what implicit bias is, rather than on repeated practice of recognizing one’s own biases and responding in non-stigmatizing ways (Burgess et al., 2017). Providers need training that teaches how to pause when noticing stigmatizing beliefs, judgments, and assumptions in order to choose a response that is productive and supportive.

One method to address stigma that has received much attention is the practice of mindfulness. Mindfulness is regarded as mental training meant to increase nonjudgmental and nonreactive awareness of the present moment (Zou et al., 2016). Mindfulness-based interventions for healthcare providers have been shown to reduce emotional exhaustion and depersonalization (Goodman and Schorling, 2012), work-related stress and burnout (Thimmapuram et al., 2017), and depression and anxiety (Johnson et al., 2015), while increasing empathy, emotional awareness, self-efficacy, and compassion (Lamothe et al., 2016). Much research on mindfulness-based interventions for healthcare providers exists; however, implementation of such evidence-based resources is limited across healthcare systems.

As the practice of mindfulness meditation has been largely embraced in the United States, it is also important to reflect on the roots of mindfulness and the benefits of understanding the commonalities between the practice of modern medicine and Buddhism. Mindfulness is only one facet of an entire system rooted in ethical conduct, developed by Siddhartha Gautama, who eventually became known as the Buddha (Bodhi, 1999). The Buddha’s teachings centered on understanding the root cause of suffering and the process of alleviating and preventing suffering, much like the intention of healthcare. Actually, the guiding moral principles of ancient Buddhism largely go in tandem with the ethical principles of modern medicine. As described by Kalra et al. (2018), the Buddhist text, Dhammapada (verse 183) guides practitioners “not to do any evil,” which aligns with the principle of non-maleficence; “to cultivate what is good,” which aligns with beneficence; and to “purify one’s mind,” which aligns with the principles of justice and autonomy. Ancient Buddhist texts point toward ethical conduct/morality as the glue for friendliness, trust, and successful relationships with self and others. The Buddha provided experientially-based guidance for upholding one’s ethical commitment and intention toward the alleviation of suffering, referred to as the Noble Eightfold Path. Briefly, the Noble Eightfold Path encompasses eight components, presented as a sequence, but better understood as “the intertwining strands of a single cable that requires the contribution of all the strands for maximum strength” (Bodhi, 1999). Also described by Bodhi (1999), the eight components can be organized according to three groups: moral discipline (which includes right speech, right action, and right livelihood); concentration (which includes right effort, right mindfulness, and right concentration); and wisdom (which includes right view and right intention). More so than “correct,” the word “right” in this context means what is “real” and “true” based on one’s own values when one’s mind is clear of unwholesome states, such as delusion, ill-will, hate, and greed (Bodhi, 1999).

Believing another person is less deserving of compassionate care can have detrimental effects when unexamined. Healthcare workers hold positions of power over others, and this puts them in a better position to initiate and facilitate a friendlier attitude toward patients. The Eightfold Path provides a specific recipe for leading interactions with value-based choices rather than reactive impulses. Whether guided by the Eightfold Path or one’s professional code of conduct, healthcare professionals have a duty to treat all patients with dignity and respect, and that may mean setting aside one’s personal beliefs in order to fulfill one’s professional oath.

## Mindfulness skills to manage “Othering Bias”

Mindfulness, like any other learned behavior, depends on *knowledge* (i.e., insight and wisdom) and *skill* (i.e., requires practice and repetition). For example, driving a car requires both knowledge about how the vehicle operates and continuous practice. Driving cannot be fully learned through books, it requires first-hand training. Mindfulness practice is similar in that it is experiential and applied, and cannot be grasped fully through theoretical and passive learning.

Continuous mindfulness practice increases one’s capacity to attend to the present moment with curiosity and openness and provides a foundation for *metacognition*, which refers to one’s “awareness of one’s own cognitive processes, including examining one’s own biases and decision-making” (Szczepanik et al., 2020). Metacognition or “thinking about thinking” (Shapiro et al., 2006) begins with understanding that one can experience thoughts and emotions without being consumed by the experience and immediately acting upon it (Shapiro et al., 2006). For example, a provider may learn a pregnant patient is using substances and have the automatic thought, “What a bad parent,” and that thought may provoke emotions such as anger and disdain. When one is fused with and controlled by emotions, one may engage reactively to alleviate emotional discomfort. Through metacognition, one can separate from their emotions, witnessing them and the impulse toward emotional reactivity, while taking the time to decide how to respond purposefully and consciously. By utilizing metacognition, mindfulness practice can help skillfully manage “othering bias” through increased mental clarity and concentration.

Metacognition, clarity, and concentration *skills* can be acquired through mindfulness meditation. This type of meditation practice centers on observing the breath while seated or lying down with eyes closed or down-gazed to limit visual distractions. As the mind inevitably wanders and becomes caught up in unprompted thoughts, one practices recognizing mind-wandering and returning focus to the breath. Repeated practice of acknowledging mind-wandering and returning attention to the breath leads to experiential knowledge of how thoughts arise and how one can respond accordingly. One can choose to let the thoughts go and bring attention back to the breath, or one can choose to follow the thought and become invested in the thought. By noticing the emotional experience of each option, one can learn which thoughts increase well-being or suffering. Mindfulness allows one to engage intentionally with the Eightfold Path.

The mindfulness practice of right view (one of the factors in the Noble Eightfold Path to end suffering) centers on the understanding of the origin of suffering and the pathway toward the cessation of suffering (Digha Nikaya 22, as cited by Bodhi, 1999) and is often viewed as the basis for the rest of the Eightfold Path. Right view may allow providers to maintain an awareness of substance use as a means of coping with suffering. Although substance use provides a “false refuge” where the relief from suffering is temporary (Groves and Farmer, 2009), it does provide an immediate “quick-fix” for discomfort.

Right view can help providers remember the person in front of them has a full and intricate biopsychosocial history, and is not defined by substance utilization. With an intention toward compassion and seeing the person behind the substance use, providers may be more likely to engage in ethical and wise behaviors, such as right speech and right effort.

The mindfulness practice of Right Speech (RSP) would be effective for developing *skills* to prevent speaking reactively as it brings the mind into a metacognitive perspective. RSP requires one to think and compose the speech before speaking, learning to consider the following guidelines (Access to Insight, 2005):

1. Is it objectively true?
2. Does it have value?
3. Is it proper within the context?
4. Is it free from the intention to prop-up one’s ego?
5. Will my speech be peaceful upon its delivery?

Metacognition, mental clarity, and concentration can be utilized to observe thoughts and mental narratives before speaking and acting. By identifying a certain mental bias and “noting” (naming) it, without harsh judgment or self-criticism, as “bias,” “othering,” or “stigmatizing,” one has the opportunity to respond in a way that honors another’s dignity and lived experience. An individual can practice noting anytime and anywhere by focusing attention on the context of their thoughts.

Right Effort (Bodhi, 1999) involves discipline to continue engaging in behaviors that align with one’s values, such as engaging in continued meditation and recognizing when bias or stigma is arising in one’s mind. Right effort may also involve taking care of oneself as a healthcare provider, as fatigue, burnout, stress, and neglecting self-care deplete one’s resources and mental capacities, making it more difficult to engage with intention. When depleted, one will likely revert to automatic behaviors. Right effort allows providers to create a place of safety and support, rather than discrimination and ostracization. A deeper awareness of mindfulness practices within the context of ethical conduct can support healthcare shifts away from criminalization toward more compassionate patient- and family-centered approaches.

## Conclusion

With the current state of the maternal health crisis in the United States, as well as the reality that perinatal substance use is a leading cause of maternal death, more comprehensive training practices in reducing stigma among providers is crucial. Stigmatizing behaviors actively prevent and discourage perinatal patients from engaging with the healthcare system. Improving healthcare services for pregnant individuals using substances cannot only be patient-facing, it must also be provider- and clinic-facing; reducing patient stigma is a top-down, systems issue. Each provider must take an honest look at their assumptions about, behaviors toward, and comments to patients. By using the experiential practices within mindfulness-based interventions and the ancient roots of mindfulness as an element of ethical conduct within Eightfold Path, providers can identify their overarching values and lead their healthcare encounters with compassion and willingness to support help-seeking community members who are experiencing suffering.

## Data availability statement

The original contributions presented in the study are included in the article/supplementary material, further inquiries can be directed to the corresponding author.

## Author contributions

TS: Conceptualization, Writing – original draft, Writing – review & editing. ST: Conceptualization, Writing – original draft, Writing – review & editing. TR: Conceptualization, Writing – review & editing. KM: Supervision, Writing – review & editing, Conceptualization.

## References

[ref1] Access to Insight (ed.) (2005). Right Speech: samma vaca. Access to Insight. Available at: http://www.accesstoinsight.org/ptf/dhamma/sacca/sacca4/samma-vaca/index.html

[ref2] Akbulut N. Razum O. (2022). Why othering should be considered in research on health inequalities: theoretical perspectives and research needs. SSM Popul. Health 20:101286. doi: 10.1016/j.ssmph.2022.101286, PMID: 36406107 PMC9672483

[ref3] Blair L. Ashford K. Gentry L. Bell S. Fallin-Bennett A. (2021). Care experiences of persons with perinatal opioid use a qualitative study. J. Perinat. Neonatal Nurs. 35, 320–329. doi: 10.1097/JPN.0000000000000597, PMID: 34726648

[ref4] Bodhi B. (1999). The Noble eightfold path: The way to the end of suffering. Access to Insight (BCBS Edition). Available at: http://www.accesstoinsight.org/lib/authors/bodhi/waytoend.html

[ref5] Bos A. E. R. Pryor J. B. Reeder G. D. Stutterheim S. E. (2013). Stigma: advances in theory and research. Basic Appl. Soc. Psychol. 35, 1–9. doi: 10.1080/01973533.2012.746147

[ref9003] Bridges K. M. (2020). Race, pregnancy, and the opioid epidemic: White privilege and the criminalization of opioid use during pregnancy. Harv. Law Rev. 133, 770–851.

[ref6] Bright V. Riddle J. Kerver J. (2022). Stigma experienced by rural pregnant women with substance use disorder: a scoping review and qualitative synthesis. Int. J. Environ. Res. Public Health 19:15065. doi: 10.3390/ijerph192215065, PMID: 36429782 PMC9690597

[ref7] Bruzelius E. Martins S. S. (2022). US trends in drug overdose mortality among pregnant and postpartum persons, 2017-2020. JAMA 328, 2159–2161. doi: 10.1001/jama.2022.17045, PMID: 36472602 PMC9856503

[ref8] Burgess A. Bauer E. Gallagher S. Karstens B. Lavoie L. Ahrens K.. (2021). Experiences of stigma among individuals in recovery from opioid use disorder in a rural setting: a qualitative analysis. J. Subst. Abus. Treat. 130:108488. doi: 10.1016/j.jsat.2021.108488, PMID: 34118715

[ref9] Burgess D. J. Beach M. C. Saha S. (2017). Mindfulness practice: a promising approach to reducing the effects of clinician implicit bias on patients. Patient Educ. Couns. 100, 372–376. doi: 10.1016/j.pec.2016.09.005, PMID: 27665499

[ref10] Denny C. H. Acero C. S. Naimi T. S. Kim S. Y. (2019). Consumption of alcohol beverages and binge drinking among pregnant women aged 18–44 years—United States, 2015–2017. MMWR Morb. Mortal Wkly. Rep. 68, 365–368. doi: 10.15585/mmwr.mm6816a1, PMID: 31022164 PMC6483284

[ref11] Earnshaw V. A. (2020). Stigma and substance use disorders: a clinical, research, and advocacy agenda. Am. Psychol. 75, 1300–1311. doi: 10.1037/amp0000744, PMID: 33382299 PMC8168446

[ref12] England L. J. Bennett C. Denny C. H. Honein M. A. Gilboa S. M. Kim S. Y.. (2020). Alcohol use and co-use of other substances among pregnant females aged 12-44 years—United States, 2015-2018. MMWR Morb. Mortality Wkly. Rep. 69, 1009–1014. doi: 10.15585/mmwr.mm6931a1, PMID: 32759915 PMC7454897

[ref9005] Goffman E. (1963). Stigma: Notes on the management of spoiled identity. Prentice Hall.

[ref13] Goodman M. J. Schorling J. B. (2012). A mindfulness course decreases burnout and improves well-being among healthcare providers. Int. J. Psychiatry Med. 43, 119–128. doi: 10.2190/PM.43.2.b, PMID: 22849035

[ref14] Groves P. Farmer R. (2009). Buddhism and addictions. Addict. Res. Theory 2, 183–194. doi: 10.3109/16066359409109142

[ref16] Harvey S. Schmied V. Nicholls D. Dahlen H. (2015). Hope amidst judgement: the meaning mothers accessing opioid treatment programmes ascribe to interactions with health services in the perinatal period. J. Fam. Stud. 21, 282–304. doi: 10.1080/13229400.2015.1110531

[ref9007] Hill J. V. Leeming D. (2014). Reconstructing ‘the alcoholic’: Recovering from alcohol addiction and the stigma this entails. Int. J. Ment. Health Addict. 12, 759–771. doi: 10.1007/s11469-014-9508-z

[ref17] Howard H. (2015). Reducing stigma: lessons from opioid-dependent women. J. Soc. Work. Pract. Addict. 15, 418–438. doi: 10.1080/1533256X.2015.1091003

[ref18] Johnson J. R. Emmons H. C. Rivard R. L. Griffin K. H. Dusek J. A. (2015). Resilience training: a pilot study of a mindfulness-based program with depressed healthcare professionals. Exp. Dermatol. 11, 433–444. doi: 10.1016/j.explore.2015.08.002, PMID: 26410675

[ref19] Kalra S. Priya G. Grewal E. Aye T. T. Waraich B. K. SweLatt T.. (2018). Lessons for the health-care practitioner from Buddhism. Ind. J. Endocrinol. Metabol. 22, 812–817. doi: 10.4103/ijem.IJEM_286_17, PMID: 30766824 PMC6330872

[ref9001] Kennedy-Hendricks A. McGinty E. E. Barry C. L. (2016). Effects of competing narratives on public perceptions of opioid pain reliever addiction during pregnancy. J. Health Pol. Pol’ & L. 41, 873–916. 10.1215/03616878-3632230 27256811

[ref9002] Kerker B. D. Leventhal J. M. Schlesinger M. Horwitz S. M. (2006). Racial and ethnic disparities in medical history taking. Ethnicity & Disease, 16, 28–34. 16599345

[ref20] Khantzian E. J. (1997). The self-medication hypothesis of substance use disorders: a reconsideration and recent applications. Harv. Rev. Psychiatry 4, 231–244. doi: 10.3109/10673229709030550, PMID: 9385000

[ref21] Ko J. Y. D’Angelo D. V. Haight S. C. Morrow B. Cox S. Salvesen von Essen B.. (2020). Prescription opioid pain reliever use during pregnancy—34 U.S. jurisdictions, 2019. MMWR Morb. Mortal Wkly. Rep. 69, 897–903. doi: 10.15585/mmwr.mm6928a1, PMID: 32673301 PMC7366850

[ref22] Lamothe M. Rondeau É. Malboeuf-Hurtubise C. Duval M. Sultan S. (2016). Outcomes of MBSR or MBSR-based interventions in health care providers: a systematic review with a focus on empathy and emotional competencies. Complement. Ther. Med. 24, 19–28. doi: 10.1016/j.ctim.2015.11.001 26860797

[ref23] Lipsett M. Wyant-Stein K. Mendes S. Berger E. Berkman E. T. Terplan M.. (2023). Addressing stigma within the dissemination of research products to improve quality of care for pregnant and parenting people affected by substance use disorder. Front. Psychol. 14:1199661. doi: 10.3389/fpsyt.2023.1199661, PMID: 37351006 PMC10282149

[ref9006] Mackiewicz Seghete K. L. M. Graham A. M. Shank T. M. Alsup S. L. Fisher P. A. Wilson A. C.. (2020). Advancing preventive interventions for pregnant women who are opioid using via the integration of addiction and mental health research. Curr. Addict. Rep. 7, 61–67. doi: 10.1007/s40429-020-00296-x 32201680 PMC7083585

[ref24] National Institute on Drug Abuse (2016). National Institute on Drug Abuse 2016–2020 strategic plan. Available at: https://nida.nih.gov/sites/default/files/2016-2020nidastrategicplan.pdf

[ref25] Nichols T. R. Welborn A. Gringle M. R. Lee A. (2021). Social stigma and perinatal substance use services: recognizing the power of the good mother ideal. Contemp. Drug Probl. 48, 19–37. doi: 10.1177/0091450920969200

[ref26] Patrick S. W. Richards M. R. Dupont W. D. McNeer E. Buntin M. B. Martin P. R.. (2020). Association of Pregnancy and insurance status with treatment access for opioid use disorder. JAMA Netw. Open 3:e2013456. doi: 10.1001/jamanetworkopen.2020.13456, PMID: 32797175 PMC7428808

[ref27] Phelan J. C. Link B. G. Dovidio J. F. (2008). Stigma and prejudice: one animal or two? Soc. Sci. Med. 67, 358–367. doi: 10.1016/j.socscimed.2008.03.022, PMID: 18524444 PMC4007574

[ref28] Racine E. Bell E. Zizzo N. Green C. (2015). Public discourse on the biology of alcohol addiction: implications for stigma, self-control, essentialism, and coercive policies in pregnancy. Neuroethics 8, 177–186. doi: 10.1007/s12152-014-9228-x

[ref9004] Roberts S. C. M. Nuru-Jeter A. (2012). Universal screening for alcohol and drug use and racial disparities in child protective services reporting. J. Behav. Health Serv. Res. 39, 3–16. doi: 10.1007/s11414-011-9247-x 21681593 PMC3297420

[ref31] Shapiro S. L. Carlson L. E. Astin J. A. Freedman B. (2006). Mechanisms of mindfulness. J. Clin. Psychol. 62, 373–386. doi: 10.1002/jclp.20237 16385481

[ref32] Stone R. (2015). Pregnant women and substance use: fear, stigma, and barriers to care. Health Justice 3:2. doi: 10.1186/s40352-015-0015-5

[ref33] Substance Abuse and Mental Health Services Administration Department of Health and Human Services (2019). Center for Behavioral Health Statistics and Quality 2019 National Survey on drug use and health: Women. Maryland (US): Substance Abuse and Mental Health Services. Available at: https://www.samhsa.gov/data/report/2019-nsduh-women

[ref34] Sweigart E. (2017). Compassion fatigue, burnout, and neonatal abstinence syndrome. Neonatal Netw. 36, 7–11. doi: 10.1891/0730-0832.36.1.7, PMID: 28137347

[ref35] Szczepanik J. E. Brycz H. Kleka P. Fanslau A. Zarate C. A. Jr. Nugent A. C. (2020). Metacognition and emotion—how accurate perception of own biases relates to positive feelings and hedonic capacity. Conscious. Cogn. 82:102936. doi: 10.1016/j.concog.2020.102936, PMID: 32416543 PMC7322564

[ref36] Terplan M. Kennedy-Hendricks A. Chisolm M. S. (2015). Article commentary: prenatal substance use: exploring assumptions of maternal unfitness. Subst. Abus. 9, 1–4. doi: 10.4137/SART.S23328, PMID: 26448685 PMC4578572

[ref37] Thimmapuram J. Pargament R. Sibliss K. Grim R. Risques R. Toorens E. (2017). Effect of heartfulness meditation on burnout, emotional wellness, and telomere length in health care professionals. J. Community Hosp. Intern. Med. Perspect. 7, 21–27. doi: 10.1080/20009666.2016.1270806, PMID: 28634520 PMC5463663

[ref38] Trost S. L. Beauregard J. Chandra G. Njie F. Berry J. Harvey A.. (2022). Pregnancy-related deaths: Data from maternal mortality review committees in 36 US states, 2017–2019. Centers for Disease Control and Prevention, US Department of Health and Human Services. Available at: https://www.cdc.gov/reproductivehealth/maternal-mortality/erase-mm/data-mmrc.html

[ref39] Volkow N. (2023). Pregnant people with substance use disorders need treatment, not criminalization. National Institute on Drug Abuse. Available at: https://nida.nih.gov/about-nida/noras-blog/2023/02/pregnant-people-substance-use-disorders-need-treatment-not-criminalization

[ref40] Webb R. Uddin N. Ford E. Easter A. Shakespeare J. Roberts N.. (2021). Barriers and facilitators to implementing perinatal mental health care in health and social care settings: a systematic review. Lancet Psychiatry 8, 521–534. doi: 10.1016/S2215-0366(20)30467-3, PMID: 33838118

[ref41] Weber A. Miskle B. Lynch A. Arndt S. Acion L. (2021). Substance use in pregnancy: identifying stigma and improving care. Subst. Abus. Rehabil. 12, 105–121. doi: 10.2147/SAR.S319180, PMID: 34849047 PMC8627324

[ref42] Zou T. Wu C. Fan X. (2016). The clinical value, principle, and basic practical technique of mindfulness intervention. Shanghai Arch. Psychiatry 28, 121–130. doi: 10.11919/j.issn.1002-0829.216060, PMID: 28638181 PMC5434297

